# Biological Mechanisms Underlying the Ultraviolet Radiation-Induced Formation of Skin Wrinkling and Sagging II: Over-Expression of Neprilysin Plays an Essential Role 

**DOI:** 10.3390/ijms16047776

**Published:** 2015-04-08

**Authors:** Genji Imokawa, Hiroaki Nakajima, Koichi Ishida

**Affiliations:** 1 Research Institute for Biological Functions, Chubu University, 1200 Matsumoto, Kasugai, Aichi 487-8501, Japan; 2 School of Bioscience and Biotechnology, Tokyo University of Technology, 1404-1 Katakura, Hachioji, Tokyo 192-0982, Japan; E-Mail: ozzy_ozzy41@yahoo.co.jp; 3 R&D-Skin Care Products Research, Kao Corporation, Tokyo 131-8501, Japan; E-Mail: ishida.koichi@kao.co.jp

**Keywords:** ultraviolet A, ultraviolet B, keratinocytes, fibroblasts, elastase, neprilysin, neutral endopeptidase

## Abstract

Our previous studies strongly indicated that the up-regulated activity of skin fibroblast-derived elastase plays a pivotal role in wrinkling and/or sagging of the skin via the impairment of elastic fiber configuration and the subsequent loss of skin elasticity. Fortunately, we succeeded in identifying human skin fibroblast-derived elastase as a previously known enzyme, neprilysin or neutral endopeptidase (NEP). We have also characterized epithelial-mesenchymal paracrine cytokine interactions between UVB-exposed-keratinocytes and dermal fibroblasts and found that interleukin-1α and granulocyte macrophage colony stimulatory factor (GM-CSF) are intrinsic cytokines secreted by UVB-exposed keratinocytes that stimulate the expression of neprilysin by fibroblasts. On the other hand, direct UVA exposure of human fibroblasts significantly stimulates the secretion of IL-6 and also elicits a significant increase in the gene expression of matrix metallo-protease(MMP)-1 as well as neprilysin (to a lesser extent), which is followed by distinct increases in their protein and enzymatic activity levels. Direct UVA exposure of human keratinocytes also stimulates the secretion of IL-6, IL-8 and GM-CSF but not of IL-1 and endothelin-1. These findings suggest that GM-CSF secreted by UVA-exposed keratinocytes as well as IL-6 secreted by UVA-exposed dermal fibroblasts play important and additional roles in UVA-induced sagging and wrinkling by up-regulation of neprilysin and MMP-1, respectively, in dermal fibroblasts.

## 1. Introduction

Our previous studies strongly indicated that the up-regulated activity of skin fibroblast-derived elastase plays a pivotal role in wrinkling and/or sagging of the skin via the impairment of elastic fiber configuration and the subsequent loss of skin elasticity. This review focuses on the later part of our long-term research project directed towards clarifying the mechanism(s) of formation of UV-induced wrinkling and sagging of the skin in an evidence-based fashion. Thus, we discuss several approaches used to search for UV-induced wrinkling or sagging mechanisms, as follows: (1) To identify the enzymatic properties of skin fibroblast-derived elastase; (2) To ask what epithelial-mesenchymal interaction is involved in the biological mechanism(s) by which the expression of skin fibroblast-derived elastase is distinctly up-regulated in the UVB-exposed skin; (3) To ask the effects of UVA radiation on human keratinocyte and human fibroblasts, leading to the increased expression of skin fibroblast-derived elastase in the UVA-exposed dermis.

## 2. Characterization and Identification of Skin Fibroblast Elastase

Although there is some evidence that skin fibroblasts synthesize an “elastase”, little is known about its specific enzyme species and its enzymatic and molecular properties because its purification up to a single band by SDS-PAGE has not been attained. Skin fibroblast-derived elastase has been reported to be a 94 kDa membrane-bound type metalloprotease with a neutral optimum pH [1,2,3,4,5,6,7,8]. Although 92, 72 kDa type IV collagenase (gelatinase) [9,10], neutrophil elastase [11], cathepsin G [12] and protease 3 [13] had been considered as candidate enzymes for skin fibroblast-derived elastase, none of those had properties which matched the characteristics of skin fibroblast-derived elastase. 

Fortunately, we were able to notice several similarities between skin fibroblast-derived elastase and NEP/ neprilysin [14] in terms of their size (97,000 Da), their membrane-bound nature and their inhibitory profiles. NEP has been reported to be an enzyme that cleaves insulin B chain from the rabbit renal pelvic membrane [15]. It is a plasma membrane-bound metalloproteinase with a molecular weight of 98,000 and a neutral optimum pH [16]. Many peptides, including enkephalin [17,18], substance P [19], bombesin-like peptide [20,21], endothelin [22,23], bradykinin [19] and others [24], have been considered as substrates for NEP. Another name for NEP, enkephalinase, reflects the fact that NEP can degrade enkephalin in association with nerves [18,25,26]. In relation to the lymph system [27,28], NEP has recently been identified as CD10, a differentiation marker of B cells and neutrophils [29]. Another name for NEP, neprilysin, has been identified as a unique physiological amyloid β peptide-degrading enzyme that cleaves the amyloid-β peptide, the abnormal misfolding and aggregation of which are implicated in neural tissue as a cause of Alzheimer’s disease [30]. Interestingly, the expression level of neprilysin in the brain is distinctly down-regulated with increasing age and in the early stages of Alzheimer’s disease during which impaired metabolism of amyloid-β peptide 3 plays a central pathological role [31,32]. However, there had been no report associating neprilysin with skin fibroblast-derived elastase. Thus, we used immunoprecipitation and Western blotting with an anti-neprilysin/NEP antibody to prove the identity of skin fibroblast-derived elastase and neprilysin [33].

**Figure 1:**
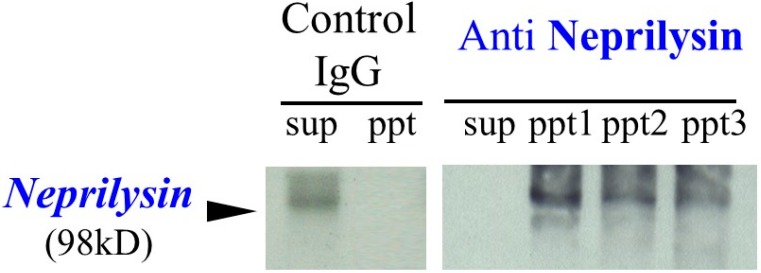
Western blotting of supernatants and precipitates following immunoprecipitation with anti-neprilysin or control IgG.

In human skin fibroblasts, immunohistochemistry with an antibody to human neprilysin exhibits positive staining on the plasma membrane but not within the nuclei [33], which indicates the existence of neprilysin in human skin fibroblasts. By immunoprecipitation with an anti-neprilysin antibody, elastase activity in extracts of human skin fibroblasts was found to co-precipitate with the anti-neprilysin antibody. Those precipitates have distinct elastase and NEP activities for a synthetic substrate and for insoluble elastin [33]. Moreover, thiorphan, a specific inhibitor of neprilysin [24,34], and phosphoramidon, a metalloproteinase inhibitor [35,36], almost completely inhibit the elastase activity in both the cell extracts and the immunoprecipitates, which strongly suggests that skin fibroblast-derived elastase is identical to neprilysin. Consistently, by western blotting of the supernatant and the precipitate following immunoprecipitation with anti-neprilysin or control IgG, human skin fibroblasts were found to contain a 98 kDa protein immunoprecipitatable with the anti-neprilysin antibody [33] (Figure 1).

After expression vectors with an inserted human neprilysin cDNA were transfected into COS-1 cells (an African green monkey kidney fibroblast-like cell line). RT-PCR analysis demonstrated that the transfected COS-1 cells express neprilysin mRNA to an extent similar to human skin fibroblasts. While extracts of non-transfected COS-1 cells have extremely low levels of neprilysin/NEP activity, transfection of the human neprilysin cDNA expression vector elicits a marked increase in neprilysin activity [33]. These findings strongly suggest that the elastase activity detectable in human skin fibroblasts can be predominantly ascribed to neprilysin.

Since the photoaging-inducible human skin fibroblast-derived elastase is identical to neuropeptide-degradable neprilysin, it is of considerable interest to determine whether there is a mechanistic link between skin photoaging and neuropeptides such as enkephalin [17,18], substance P [19] and bombesin-like peptide [20,21]. Neprilysin is well known to play an essential role in the regulation of neurotransmission [16] and in the neuroimmunocutaneous system [37,38]. In UV-exposed skin, it seems likely that the up-regulated expression of neprilysin is associated with the degradation of a variety of neuropeptides, leading to the down-regulation of neuropeptide function as a regulatory factor of inflammation [39] in concert with the elastin fiber degeneration that results in wrinkling or sagging of the skin.

## 3. Biological Mechanism Involved in the UVB-Induced Up-Regulation of Neprilysin Expression in Human Skin Fibroblasts

The biochemical identification of skin fibroblast-derived elastase as the previously known enzyme neprilysin provided an incentive for studies to clarify the mechanism(s) at the gene, protein and enzymatic levels by which UVB irradiation stimulates the expression of neprilysin in the dermis. Two possible mechanisms underlying the stimulated activity of neprilysin are as follows; (1) UVB exposure directly triggers dermal fibroblasts to increase their expression of neprilysin; and/or (2) UVB exposure causes epidermal keratinocytes to stimulate the secretion of cytokines that penetrate into the dermis and trigger dermal fibroblasts to stimulate the expression of neprilysin at the transcriptional, translational and enzymatic levels. 

## 4. Effect of Direct Exposure of Human Dermal Fibroblasts to UVB on Neprilysin Protein and Activity

To test the first hypothesis, we asked whether direct UVB exposure of human skin fibroblasts stimulates the expression of neprilysin at the protein and enzymatic levels [40] because it is possible for UVB to penetrate the most upper part of the dermis. When human skin fibroblasts were exposed to UVB at doses of 40 or 80 mJ/cm^2^, the neprilysin protein level assessed by Western blotting analysis was found to be significantly down-regulated at 72 h post-irradiation in a dose-dependent manner [40]. Consistently, UVB irradiation at doses of 40 or 80 mJ/cm^2^ significantly attenuated the enzymatic activity of elastase in human skin fibroblasts at 72 h post-irradiation. Thus, it is likely that direct UVB exposure of human skin fibroblasts does not elicit the up-regulated expression of neprilysin by which the elastic fiber network is deteriorated, leading to the loss of skin elasticity in the UVB-exposed and wrinkled skin.

## 5. Biological Mechanisms Underlying the Up-Regulation of Neprilysin in Dermal Fibroblasts by UVB-Exposed Epidermal Keratinocytes

**Figure 2:**
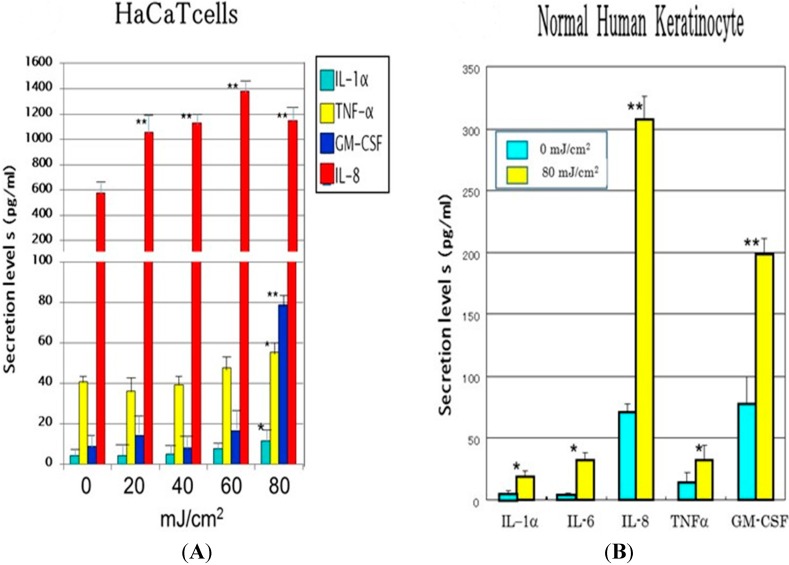
Secretion levels of cytokines (pg/mL) in the medium from HaCaT cells or HPK 72 h after UVB irradiation at 0–80 mJ/cm^2^. (**A**) HaCaT cells; (**B**) HPK. Cultured HaCaT cellsor HPK were exposed to UVB at the indicated doses and secretion levels of cytokines were measured by ELISA kit at 72 post-irradiation. Values are means ± S.D. derived from three independent experiments. ** *p* < 0.01, * *p* < 0.05 (*vs.* 0 mJ/cm^2^).

To test the second hypothesis, when human primary keratinocytes or HaCaT cells (human immortalized keratinocytes) were exposed to UVB at doses of 0–80 mJ/cm^2^ and cytokines secreted into the conditioned medium were measured by ELISA kits, secreted levels of IL-1α, IL-6, IL-8 and GM-CSF were significantly increased at 72 h post-irradiation (Figure 2). In a time course study, while the secretion of IL-6 and IL-8 was significantly increased at 5 h post-irradiation with a plateau at 20 h post-irradiation, that of IL-1α, GM-CSF and TNFα began to significantly increase at 10 h post-irradiation with a plateau at 20 h post-irradiation [40].

We next used two different approaches to characterize the epithelial-mesenchymal interaction between UVB-exposed epidermal keratinocytes and dermal fibroblasts that leads to the increased expression of neprilysin by fibroblasts in the dermis. While one method used conditioned medium from UVB-exposed keratinocytes to measure their stimulatory effect on neprilysin expression in fibroblasts, the other method utilized a co-culture system in which the two cell populations were co-cultivated in different compartments that are physically separated, but can communicate via paracrine signaling through the pores of a membrane. Using that second method, we measured the stimulatory effect of UVB-exposed keratinocytes on neprilysin expression by co-cultured fibroblasts.

**Figure 3:**
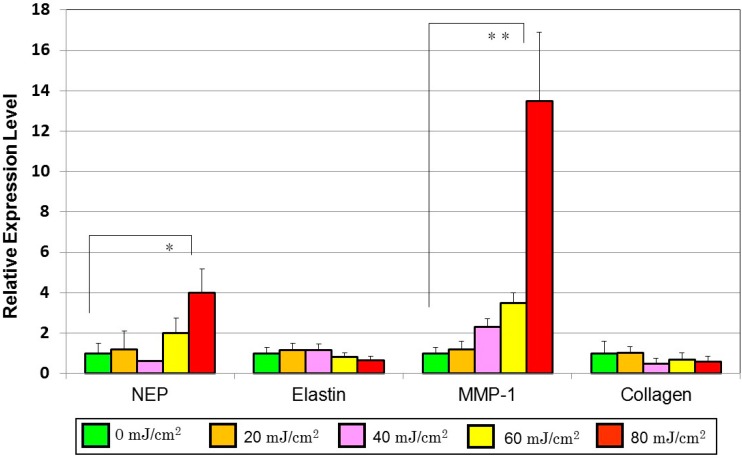
Effects of conditioned medium from UVB-exposed human primary keratinocytes on mRNA levels of neprilysin, elastin, collagenase I (MMP-1) and collagen in human dermal fibroblasts as revealed by real-time RT-PCR analysis. The conditioned medium from UVB-exposed keratinocytes was added at a 1:1 dilution to fibroblasts to measure expression levels of each mRNA at 4 h post-incubation. Values are means ± S.D. derived from three independent experiments. * *p* < 0.05, ** *p* < 0.01.

We first asked whether conditioned medium from UVB-exposed human keratinocytes stimulates the expression of genes encoding matrix proteins or MMPs in human dermal fibroblasts. We found that the conditioned medium from UVB-exposed human primary keratinocytes increased the levels of mRNAs encoding neprilysin and collagenase type I (MMP-1) (Figure 3). In contrast, levels of mRNAs encoding elastin as well as collagen were slightly attenuated at several hours post-incubation. When human fibroblasts were co-cultured with UVB-exposed human primary keratinocytes, UVB irradiation significantly up-regulated neprilysin mRNA levels at 48 h post-irradiation in human fibroblasts [40].

We compared the *in vivo* profiles of matrix proteins and MMPs in UVB-exposed and wrinkled skin and the *in vitro* cellular effects of conditioned medium from UVB-exposed human keratinocytes on gene expression patterns in human fibroblasts. It had been shown that, while there is a down-regulated expression of elastin mRNA, neprilysin and MMP-1 mRNAs are markedly up-regulated [41]. That analysis revealed that the effects of the conditioned medium on human fibroblasts mimics the *in vivo* situation in wrinkled skin despite the fact that the frequency of UVB exposure differs greatly between the *in vitro* and *in vivo* situations. This suggested that the enhanced activity of neprilysin or MMP-1 in UVB-irradiated skin [42,43] is mediated by basement membrane-permeable soluble factors secreted by UVB-exposed human keratinocytes. Taken together, the sum of available evidence supports our hypothesis for the mechanism of wrinkle formation by which cytokines are released by epidermal keratinocytes following UVB irradiation, triggering dermal fibroblasts to stimulate their expression of neprilysin. The enhanced neprilysin /NEP activity then results in the deterioration of the 3-dimensional architecture of elastic fibers, reducing skin elasticity, and eventually leading to the formation of wrinkling and/or sagging of the skin.

**Figure 4:**
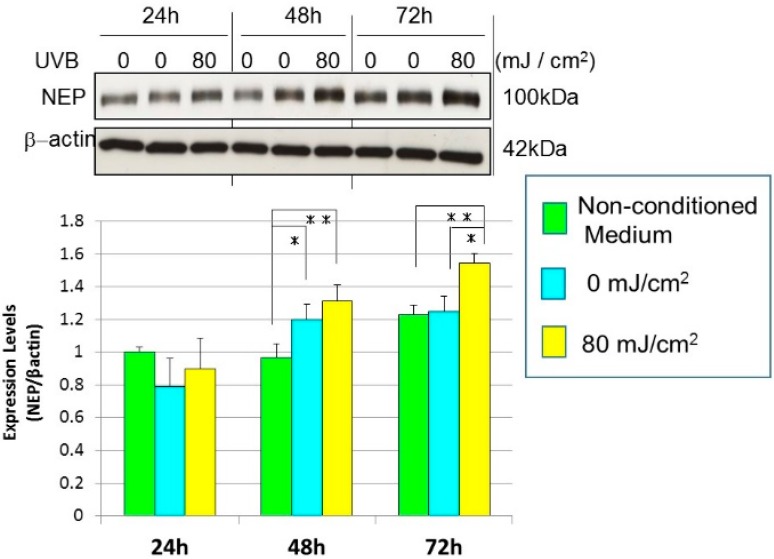
Effects of conditioned medium from UVB-exposed human primary keratinocytes on neprilysin/NEP protein in human fibroblasts. Neprilysin protein levels were measured by Western blotting analysis 24, 48 and 72 h after the addition of conditioned medium (CM) from UVB (0 or 80 mJ/cm^2^)-exposed HPKs or non-conditioned fresh DMEM medium (NCM) at a 1:1 dilution to human fibroblasts. Representative immunoblots from three independent experiments are shown. Values are means ± S.D. derived from the three independent experiments. * *p* < 0.05, ** *p* < 0.01.

**Figure 5:**
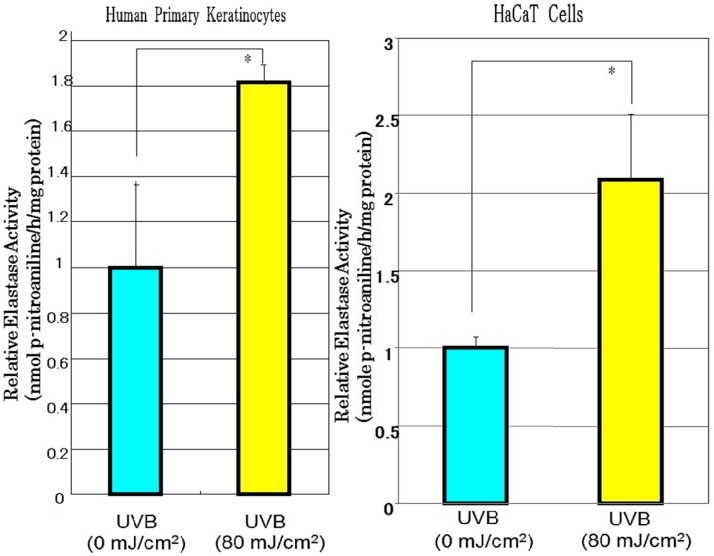
Effects of conditioned medium from UVB-exposed human primary keratinocytes or HaCaT cells on neprilysin activity in human fibroblasts. Neprilysin activity levels were measured by enzymatic assay using *N*-succinyl-*tri*-alanyl-*p*-nitroaniline (STANA) 72 h after the addition of the conditioned medium at a 1:1 dilution to human fibroblasts. The release of p-nitroaniline was measured by absorbance at 405 nm and enzymatic activity was expressed as nmol STANA/h per μg of protein to calculate as relative elastase activity. Values are means ± S.D. derived from three independent experiments. * *p* < 0.05.

Since mRNA expression levels for neprilysin in human fibroblasts were distinctly stimulated by the conditioned medium from UVB-exposed human keratinocytes, we next determined whether the conditioned medium stimulates neprilysin protein levels in human fibroblasts. Western blotting analysis for neprilysin protein revealed that when the conditioned medium from HaCaT cells exposed to UVB at a dose of 80 mJ/cm^2^ was added at a 1:1 dilution to human fibroblasts, neprilysin protein levels were markedly stimulated at 72 h post-incubation compared with the mock irradiation controls (Figure 4A). Western blotting of human fibroblasts incubated with conditioned medium from UVB-exposed human primary keratinocytes demonstrated that neprilysin protein levels in human fibroblasts are significantly increased at 48 or 72 h post-incubation (Figure 4B). Since the neprilysin mRNA and protein levels in human fibroblasts were significantly stimulated by the conditioned medium from UVB-exposed human keratinocytes, we next determined whether the conditioned medium stimulates the enzymatic activity of neprilysin in human fibroblasts. Enzymatic assays for neprilysin revealed that when the conditioned medium obtained from UVB-exposed HaCaT cells or from UVB-exposed human primary keratinocytes was added at a 1:1 dilution to human fibroblasts, the enzymatic activity of neprilysin was significantly stimulated at 72 h post-incubation compared with the mock-irradiation controls (Figure 5).

Similarly, western blotting and enzyme assays of human fibroblasts co-cultured with UVB-exposed human primary keratinocytes demonstrated that levels of neprilysin protein in human fibroblasts are significantly increased at 48 h post-incubation [40]. Enzymatic assay of human fibroblasts co-cultured with the UVB-exposed human primary keratinocytes also demonstrated that neprilysin enzymatic levels in human fibroblasts are significantly increased at 48 h post-incubation [40]. The sum of these findings indicates that paracrine factors are secreted by UVB-exposed human keratinocytes that penetrate into the dermis to trigger dermal fibroblasts to stimulate neprilysin activity.

## 6. Identification of Paracrine Cytokines Responsible for UVB-Induced Up-Regulation of Neprilysin Expression in Human Fibroblasts

To determine which cytokine(s) secreted from human keratinocytes following UVB irradiation is/are responsible for the increased expression of neprilysin, we compared the expression of neprilysin (measured by real-time RT-PCR) and levels of cytokines released into the medium by UVB-exposed human primary keratinocytes at different UVB doses [41]. There were close correlations (*R*^2^ = 0.949 and 0.947) between the secreted levels of IL-1α and GM-CSF, respectively, and the expression of neprilysin mRNA, but there was no correlation with TNFα, IL-8 or endothelin-1. This suggested that both IL-1α and GM-CSF play roles in the stimulated expression of neprilysin, although additional studies using neutralizing antibodies were required to reach a final conclusion. 

## 7. A Neutralizing Antibody Abrogates the Stimulated Neprilysin Activity

**Figure 6:**
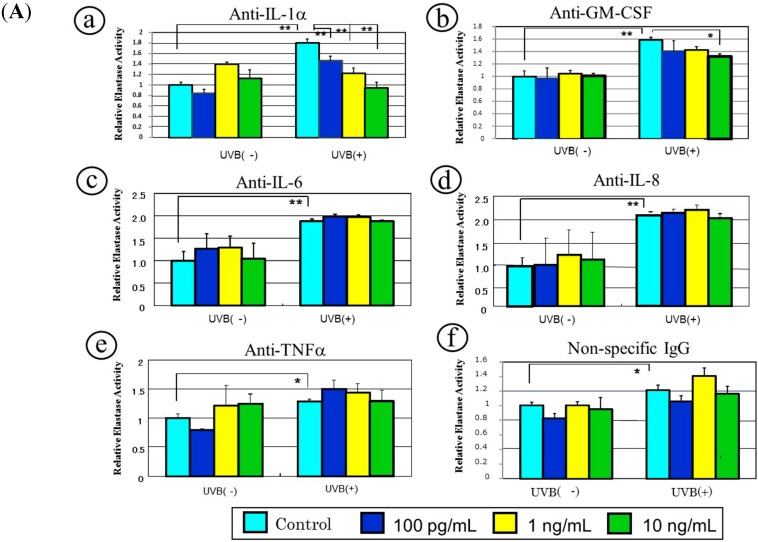

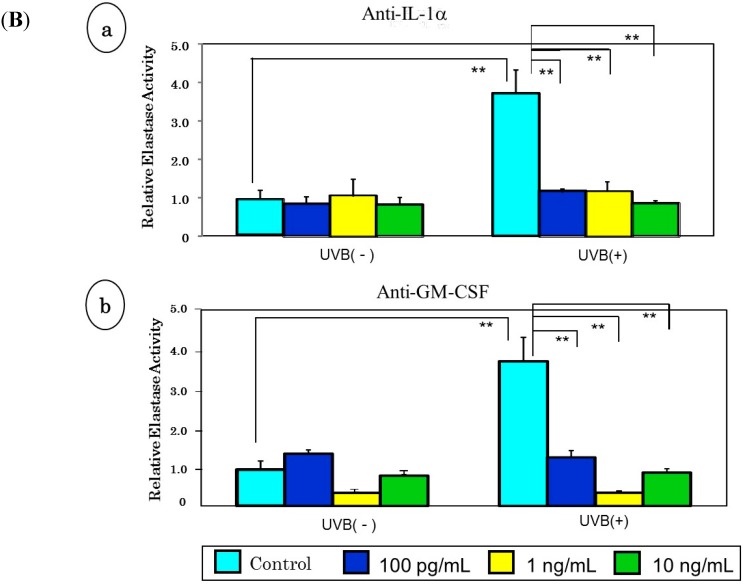
A neutralizing antibody abrogates the stimulated neprilysin activity. (**A**) HaCaT cells, (**a**) anti-human IL-1α antibody; (**b**) anti-human GM-CSF antibody; (**c**) anti-human IL-6 antibody; (**d**) anti-human IL-8 antibody; (**e**) anti-human TNFα antibody; (**f**) non-specific mouse IgG; (**B**) human primary keratinocytes, (**a**) anti-human IL-1α antibody; (**b**) anti-human GM-CSF antibody. Antibodies as well as non-specific mouse IgG were incubated at the indicated concentrations with the two-fold diluted conditioned medium. After replacement with the prepared conditioned medium, human fibroblasts were maintained in culture for 72 h. Lysates of human fibroblasts were then assessed for neprilysin activity using STANA. Values are means ± S.D. derived from 3 independent experiments. * *p* < 0.01, ** *p* < 0.05.

Since human fibroblasts cultured with the conditioned medium from UVB-exposed human primary keratinocytes significantly increased the expression of neprilysin at the transcriptional, translational and enzymatic levels, we attempted to identify the cytokines responsible for the stimulated expression of neprilysin by neutralizing experiments with antibodies to cytokines secreted in the conditioned medium. When conditioned medium obtained from UVB- or non-exposed HaCaT cells was treated with neutralizing antibodies to IL-1α, GM-CSF, IL-6, TNFα or IL-8 or with a non-specific mouse IgG, the stimulation of neprilysin activity was markedly abrogated by the anti-IL-1α and anti-GM-CSF antibodies but not by anti-IL-6, anti-IL-8 or anti-TNFα antibody or the non-specific mouse IgG (Figure 6A). When conditioned medium obtained from UVB- or non-exposed human primary keratinocytes was treated with anti-IL-1α or anti-GM-CSF antibodies, the stimulation of neprilysin activity was markedly abrogated for UVB-exposed human primary keratinocytes but not for non-exposed cells by anti-IL-1α and by anti-GM-CSF antibodies (Figure 6B).

As a second approach, we used a co-culture system to evaluate the neutralizing effects of several antibodies to cytokines or a chemokine secreted by UVB-exposed human keratinocytes on enhanced neprilysin activity in human fibroblasts [40]. Those results revealed that among the neutralizing antibodies tested (anti-IL-1α, anti-GM-CSF, anti-IL-6, anti-IL-8 and anti-TNFα), the anti-IL-1α and anti-GM-CSF (but not anti-IL-6, anti-IL-8 or anti-TNFα) antibodies significantly abrogated the enhanced activity of neprilysin in human fibroblasts after the co-culture with UVB-exposed human keratinocytes. The sum of these findings strongly suggests that IL-1α and GM-CSF are intrinsic keratinocyte-derived cytokines associated with the up-regulated expression of neprilysin. Those two antibodies also reduced the activity of neprilysin after the co-culture with non-exposed HaCaT cells. These findings indicate that the co-culture with non-exposed HaCaT cells also contains IL-1α and GM-CSF at concentrations sufficient to stimulate neprilysin activity in human skin fibroblasts, although to a lesser extent than those in the UVB-exposed cells.

Of considerable interest in the co-culture experiments using neutralizing antibodies is the fact that the addition of anti-IL-1α or anti-GM-CSF at 12 h post-irradiation did not abolish the up-regulated activity of neprilysin although their addition at 0 h post-irradiation elicited significant preventive effects on the enhanced activity of neprilysin [40]. The lack of a neutralizing effect by the later timing of addition suggests that the major release of IL-1α and GM-CSF by UVB-exposed human keratinocytes had already proceeded at 12 h post-irradiation. 

## 8. A Neutralizing Antibody Abrogates the Stimulated Neprilysin Protein

**Figure 7:**
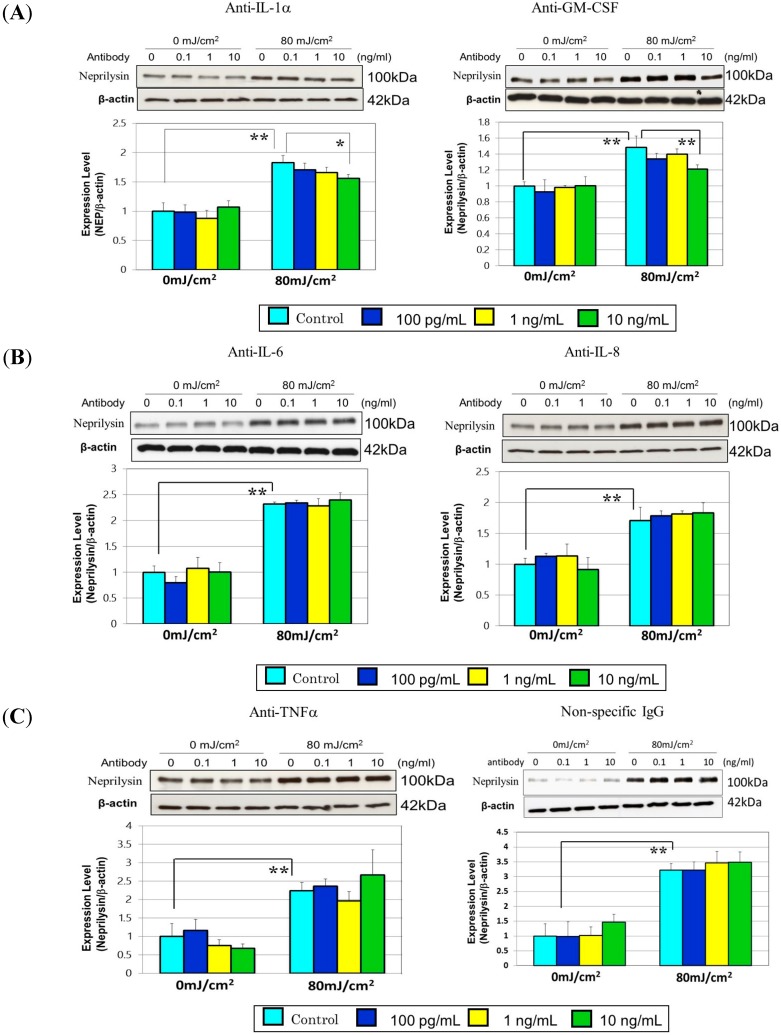
A neutralizing antibody abrogates the stimulated neprilysin (NEP) protein. (**A**) anti-human IL-1α antibody (**left**): anti-human GM-CSF antibody (**right**); (**B**) anti-human IL-6 antibody (**left**): anti-human IL-8 antibody (**right**); (**C**) anti-human TNFα antibody (**left**): non-specific mouse IgG (**right**). Antibodies as well as non-specific IgG were added at the indicated concentrations to the two-fold diluted conditioned medium. After replacement with the prepared conditioned medium, human fibroblasts were maintained in culture for 72 h. Lysates of human fibroblasts were then subjected to Western blotting analysis. Values are means ± S.D. derived from three independent experiments. ** *p* < 0.01, * *p* < 0.05.

When conditioned medium obtained from UVB- or non-exposed HaCaT cells was incubated with antibodies to IL-1α, GM-CSF, IL-6 and IL-8 or with non-specific mouse IgG, the elevated level of neprilysin protein was significantly abolished by antibodies to IL-1α and GM-CSF, but not by IL-6, IL-8, TNFα antibodies or by a non-specific mouse IgG (Figure 7).

## 9. Effects of Cytokines on the Expression of Neprilysin in Human Fibroblasts at the Gene, Protein and Enzymatic Levels 

**Figure 8:**
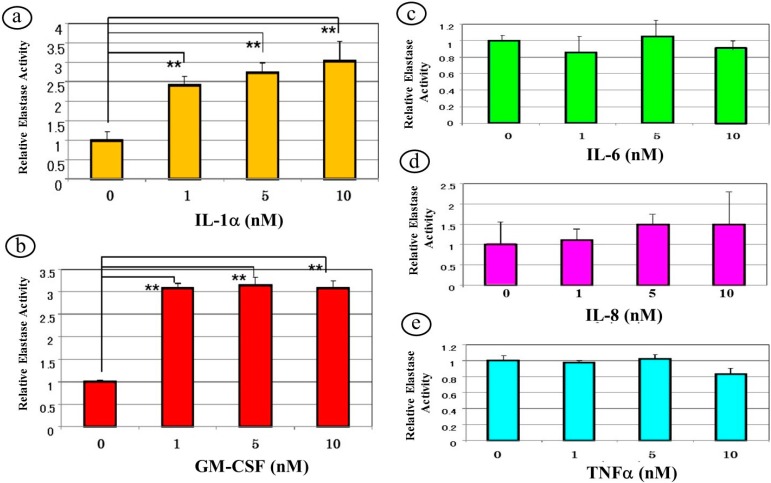
Effects of cytokines on the enzymatic activity of skin fibroblast elastase in HF as revealed by enzymatic assay using STANA. Elastase activity levels were measured by enzymatic assay using STANA 72 h after the addition of cytokines to cultured HF at the indicated concentrations. Values are means ± S.D. derived from three independent experiments. IL-1α (**a**); GM-CSF (**b**); IL-6 (**c**); IL-8 (**d**); TNFα (**e**). ** *p* < 0.01.

To determine whether the neutralizing effects of the antibodies to IL-1α or GM-CSF are associated with their ability to accentuate the expression of neprilysin in human fibroblasts, we evaluated the effects of the corresponding cytokines on the expression of neprilysin at the gene, protein and enzymatic levels. Those results demonstrated that while IL-1α, GM-SCF and TNFα, but not IL-6 or IL-8, have the ability to significantly up-regulate the expression of neprilysin mRNA in human fibroblasts, the addition of IL-1α or GM-CSF, but not IL-6, TNFα or IL-8 increases the protein levels of neprilysin [40]. Although TNFα has distinct effects at the gene and protein levels, these findings indicate that IL-1α and GM-SCF exclusively have the potential to stimulate neprilysin expression in skin fibroblasts and antibodies to those two cytokines are specifically associated with neutralizing effects on the enhanced neprilysin activity in human fibroblasts co-cultured with UVB-exposed human keratinocytes. We next dissected the ability of secreted cytokines to stimulate the enzymatic activity of neprilysin in human fibroblasts. When human fibroblasts were treated with IL-1α, GM-CSF, IL-6 or IL-8 at 0, 1, 5 and 10 nM, the enzymatic activity of neprilysin was significantly stimulated over 72 h by incubation with IL-1α or GM-CSF, but not by IL-6, IL-8 or TNFα (Figure 8).

## 10. Mechanistic Comparison of Neprilysin/Elastin Linkage with MMP-1/TbetaRII/Collagen I Linkage Leading to UVB Radiation-Induced Wrinkling and Sagging

As for biological mechanisms previously reported for UVB radiation-induced wrinkling and sagging, MMP-1 has been reported to be elevated in UVB-radiated skin, which is associated with the degradation of collagen I [44]. Excessive levels of MMP-1 can impair the structural integrity of the dermis, resulting in photoaging, which is characterized by increased coarse wrinkles [45,46,47,48]. UVB radiation also impairs the TGF-β/Smad pathway by down-regulation of the TGF-β type II receptor (TbetaRII) in dermal fibroblasts. This loss of TbetaRII prevents the downstream activation of Smad2/3 by TGF-β, thereby reducing the expression of type I procollagen [49]. In general, it has been believed that such down-regulated levels of collagen I in the dermis results in the formation of UVB-induced wrinkling [44,47,50]. However, there have been no reports to quantitatively or qualitatively associate collagen I deficiency with skin elastic properties, micro-structural features of collagen fibers in the dermis, as well as wrinkling or sagging formation. On the contrary, our studies focusing on skin fibroblast-derived elastase as a causative factor for reducing skin elastic properties more clearly demonstrated that up-regulated activity of dermal fibroblast-derived neprilysin by keratinocyte-derived cytokines plays an essential role in the alteration of elastic fiber network in the UVB-repetitively exposed skin, which results in the formation of wrinkling rather than sagging via attenuated skin elasticity.

## 11. Mechanistic Study of UVA-Induced Formation of Wrinkling and Sagging

### Effects of UVA Irradiation on Cytokine Secretion by Keratinocytes or Fibroblasts and on the Expression of Metalloproteinases by Fibroblasts

**Figure 9:**
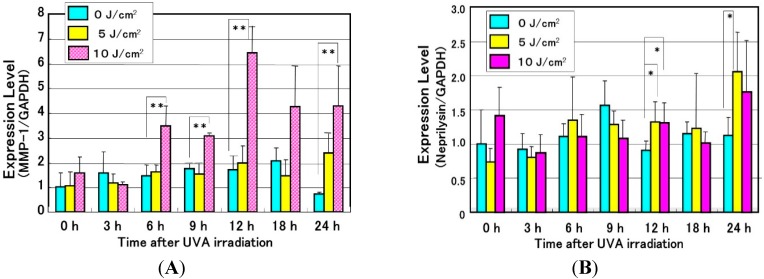
Effects of UVA radiation on mRNA levels of neprilysin (NEP) (**A**) and MMP-1 (**B**) in human dermal fibroblasts as revealed by real-time RT-PCR analysis. *n* = 5 * *p* < 0.05; ** *p* < 0.01.

**Figure 10:**
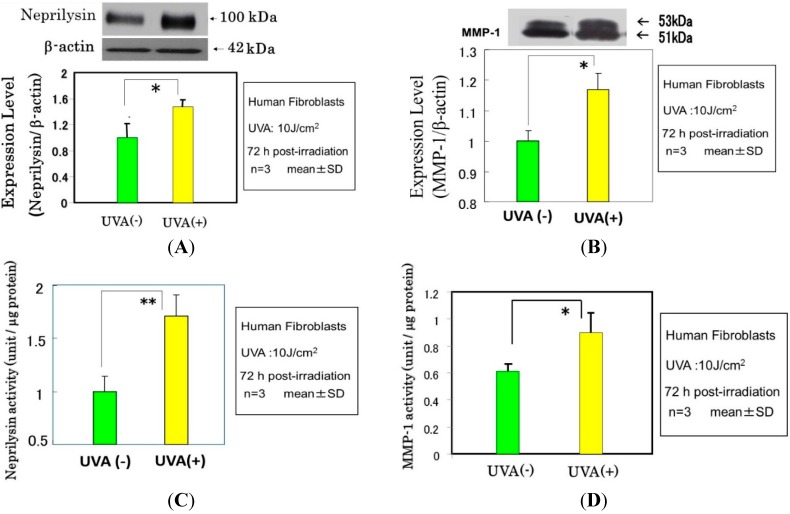
Effects of UVA radiation on the protein and activity levels of neprilysin (NEP) (**A**,**C**) and MMP-1 (**B**,**D**) in cultured human skin fibroblasts as revealed by western blotting analysis. * *p* < 0.05, ** *p* < 0.01.

**Figure 11:**
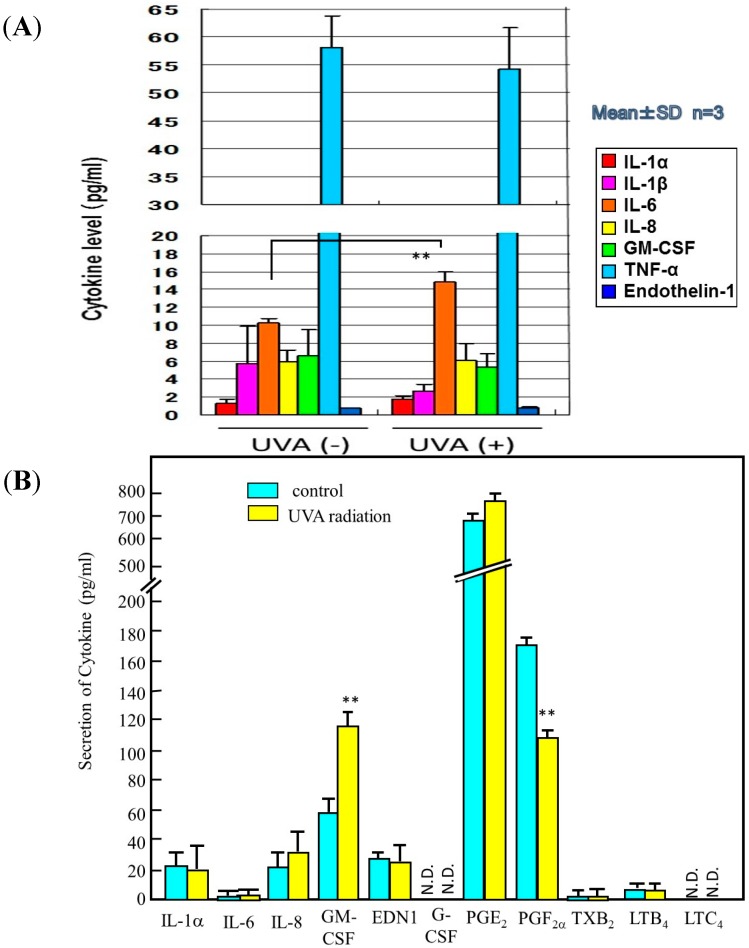
Secretion levels of cytokines (pg/mL) in the medium of cultured human dermal fibroblasts (**A**) and in the medium of cultured human primary keratinocytes (**B**) 72 h after UVA irradiation with 10 J/cm^2^. EDN1: endothelin-1, G-CSF: granulocyte-colony stimulatory factor. ** *p* < 0.01 *vs.* UVA- or control, N.D.: not detectable.

Since repetitive exposure of the skin to UVA radiation elicits sagging more frequently than wrinkling, it is likely that there are mechanisms involved other than the effects on epidermal keratinocytes to secrete cytokines. It is known that UVA irradiation penetrates into the dermis to directly affect dermal fibroblasts. As for the biological mechanism(s) involved in UVA-induced formation of sagging and wrinkling, it seems reasonable to assume that UVA-exposed dermal fibroblasts are stimulated to express neprilysin and/or MMP-1, leading to the degeneration of elastic fibers or collagen I fibers, respectively. In this respect, we found that UVA radiation elicited a significant increase in the gene expression of MMP-1 as well as neprilysin (to a lesser extent) in human fibroblasts, which was followed by distinct increases in their protein and enzymatic activity levels (Figure 9 and Figure 10) [51]. Further, analysis of the UVA-induced release of cytokines by human fibroblasts revealed that UVA irradiation significantly stimulates only the secretion of IL-6 among the cytokines tested (Figure 11A). In contrast, UVA exposure at a dose of 10 J/cm^2^, which is nearly equivalent to the average daily dose exposed to the skin during a summer day, causes human keratinocytes to stimulate the secretion of IL-6, IL-8 and GM-CSF (Figure 11B) [45,52]. These findings suggest that GM-CSF secreted by UVA-exposed keratinocytes as well as IL-6 secreted by UVA-exposed dermal fibroblasts also play important and additional roles in UVA-induced sagging and wrinkling by up-regulation of neprilysin and MMP-1, respectively, in dermal fibroblasts. Of course, because the potential to stimulate the secretion of IL-6 or GM-CSF by keratinocytes is markedly stronger by UVB than UVA, in an *in vivo* sunlight exposed-situation, the UVB effect generally overcomes the UVA effect on keratinocyte-derived cytokine-induced accentuation of the expression of neprilysin by dermal fibroblasts. In contrast, as far as the direct effect on dermal fibroblasts is concerned, UVA seems to play a predominant role in stimulating the expression of neprilysin/NEP by fibroblasts. On the other hand, the up-regulated activity of MMP-1 elicited in UVA-exposed dermal fibroblasts is predominantly associated with sagging of the skin via the diminished levels of collagen I fibers.

## 12. Conclusions

**Figure 12:**
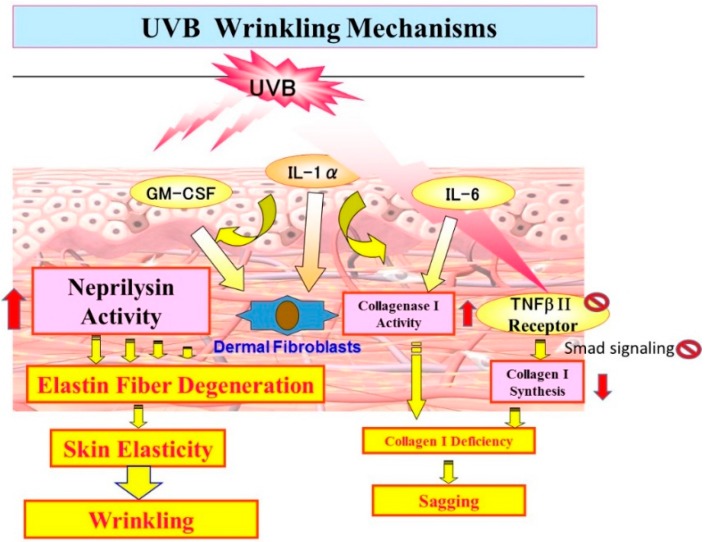
UVB wrinkling mechanisms.

**Figure 13:**
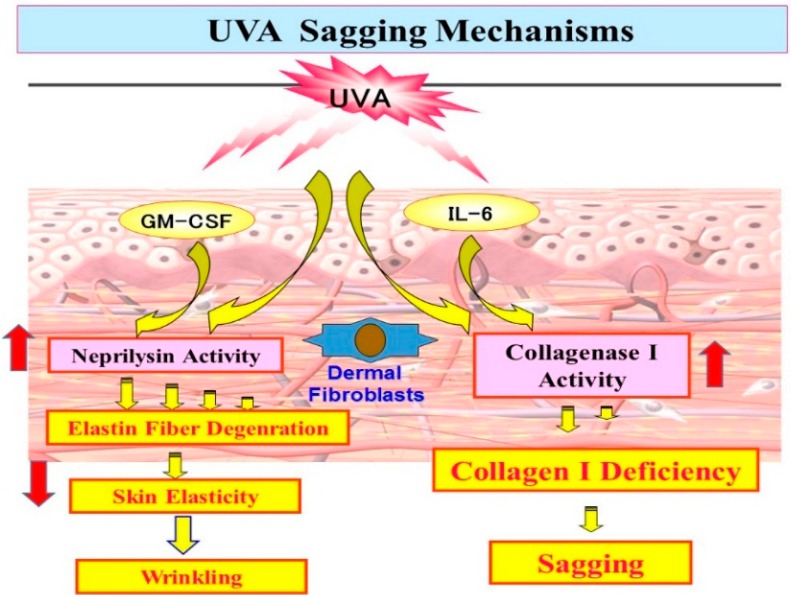
UVA sagging mechanisms.

Based on the long term research studies described above, we propose UV-induced wrinkling and sagging mechanisms as follows. As shown in Figure 12, repetitive UVB exposure causes keratinocytes to secrete IL-1α, which triggers GM-CSF secretion in an autocrine fashion. The secreted IL-1α and GM-CSF penetrate to the dermis and trigger fibroblasts to stimulate their expression of neprilysin, which then cleaves elastic fibers surrounding the fibroblasts, leading to deterioration of the elastic fiber network. The combination with the deficiency of collagen fibers [44,45,46,47,48,49,50] (to a lesser extent than deranged elastin fibers) results in a loss of skin elasticity and in turn predominately leads to wrinkling of the skin rather than sagging. On the other hand, as shown in Figure 13, repetitive UVA exposure causes keratinocytes to secrete GM-CSF to a lesser extent than UVB exposure, which triggers fibroblasts in a similar way to UVB. Further and more importantly, UVA penetrates into the dermis to directly stimulate the gene expression of MMP-1 to a greater extent than neprilysin by dermal fibroblasts and the secretion of IL-6, leading predominantly to sagging of the skin rather than wrinkling via the degradation of collagen I fibers by the enhanced activity of MMP-1. Since there is a discrepancy between the induction levels of protein and enzymatic activities of neprilysin by UV radiation and cytokines, it seems reasonable to assume that, like MMP-1, neprilysin undergoes post-translational modification to become functional. Further study on the post-translational modification of neprilysin is required for a better understanding of the biological mechanisms underlying UV radiation-induced wrinkling and sagging.

## 13. Perspectives

These newly uncovered biological mechanisms involved in the UV-induced wrinkling and sagging of the skin led us to develop a new screening system by which anti-wrinkling or sagging agents can be selectively evaluated as follows:

1. A system to determine the inhibitory effects of several candidates on IL-1α or GM-CSF-stimulated elastase activity on human fibroblasts.
2. A system to determine the inhibitory effects of several candidates on enhanced elastase activity by the conditioned medium from UVB-exposed human keratinocytes on human fibroblasts.
3. A system to determine the inhibitory effects of several candidates on enhanced elastase activity in human fibroblasts co-cultured with UVB-exposed human keratinocytes.

## Abbreviations

UVB: ultraviolet B
UVA: ultraviolet A
NEP: neutral endopeptidase
IL: interleukin
GM-CSF: granulocyte macrophage colony stimulatory factor
TNF: tumor necrosis factor
MMP-1: matrix metallo-protease-1
